# Self-care capacity of informal caregivers of older adults with dementia: quasi-experimental study*

**DOI:** 10.1590/1518-8345.7715.4677

**Published:** 2025-10-27

**Authors:** Daniela Luzia Zagoto Agulho, Annelita Almeida Oliveira Reiners, Rosemeiry Capriata de Souza Azevedo, Adriana Delmondes de Oliveira, Carla Rafaela Teixeira Cunha, Amanda Cristina de Souza Andrade, Joana Darc Chaves Cardoso, Tiago Reboulas Mazza

**Affiliations:** 1 Universidade Federal de Mato Grosso, Faculdade de Enfermagem, Cuiabá, MT, Brazil; 2 Faculdade Fasipe Cuiabá, Departamento de Enfermagem, Cuiabá, MT, Brazil

**Keywords:** Caregivers, Dementia, Self Care, Self Efficacy, Education, Nursing Education

## Abstract

to assess the effectiveness of a self-efficacy-based educational intervention to increase the self-care capacity of informal caregivers of older adults with dementia.

quasi-experimental study, with pre- and post-intervention assessment, without control group. Participants included 17 informal caregivers of older adults with dementia. The intervention had three stages: Pre-intervention with data collection to characterize the caregivers, measurement of overload, self-care capacity, and perceived self-efficacy; Intervention using four videos produced by the researcher based on the four self-efficacy belief sources of Bandura’s Social Cognitive Theory; Post-intervention with new measurement of self-care capacity. We performed descriptive analyses, normality test and non-parametric Wilcoxon Rank-Sum test and paired t-test, considering a significance level of 0.05. The research was approved by the Research Ethics Committee, under opinion 4,622,301, and was registered in the Brazilian Registry of Clinical Trials – RBR-7gfvn3y.

the comparative analysis of the effect of the educational intervention on self-care capacity found increased post-intervention mean value (102.11) in relation to the pre-intervention mean value (95.00), with statistical significance (p=0.029).

the self-efficacy-based educational intervention was effective in increasing self-care capacity for informal caregivers of older adults with dementia.

## Highlights

**(1)** The production of educational videos is valid and effective in nursing interventions. **(2)** Self-efficacy can improve self-care for caregivers of older adults with dementia. **(3)** The video-based intervention was effective in improving caregiver self-care. **(4)** Nursing interventions can promote self-care for caregivers of older adults.

## Introduction

The World Health Organization (WHO) estimated that, in 2021, approximately 55 million people worldwide had dementia, and projections indicate that this number, by 2050, could exceed 130 million^(1)^. Of the 10 million new cases reported annually, six million occur in low- and middle-income countries^(2)^. In Brazil, it was estimated that, in 2019, 1.85 million Brazilians lived with Alzheimer’s disease (AD) and other forms of dementia^(3)^. AD is the most prevalent, representing approximately 70% of cases^(4)^.

As an irreversible and progressive syndrome, dementia causes mental, physical, and social losses to patients and requires, over time, the care of an informal caregiver (IC): a person, usually a family member, who, motivated by affective, emotional, or parental bonds, assumes total or partial responsibility for the unpaid care of a dependent older family member^(5)^.

Brazil still lacks specific sociodemographic data on people who play the role of IC of older adults affected by dementia. It is only known that the number of family members who provide care to older adults in the country is growing, because, according to data from the 2020 census^(6)^, the number of family members who played this role increased from 3.7 million in 2016 to 5.1 million in 2019. A survey with paid and unpaid caregivers from several countries, including Brazil^(7)^, showed that most ICs are women, of different ethnicities and secondary educational level. In the United States, in 2023^(8)^, more than 11 million people cared for older adults affected by Alzheimer’s disease and other forms of dementia.

Commonly, providing care to people with dementia has a negative impact on the lives of ICs^(9)^, and can affect their physical, psychological, emotional, and social health, in addition to generating overload^(8-9)^, compromising the quality of the care provided to older adults and the care that caregivers dedicates to themselves, that is, self-care (SC)^(10)^.

The WHO^(11)^ defines SC as the ability of people to promote their health and prevent diseases or disabilities. SC is considered the main factor for people’s physical and mental well-being^(12)^. It is a universal practice necessary to maintain healthy relationships with oneself and with others, contributing directly to the general well-being and the prevention of diseases or complications, involving aspects such as health, thoughts, emotions, and attitudes^(13)^. By dedicating time and attention to personal care, they can strengthen their ability to deal with stress and the demands of daily routine and even with overload^(14)^. In the case of caregivers, SC, in addition to favoring well-being, provides a higher quality of the care provided to older adults with dementia^(15)^. Accordingly, there are recommendations on the need to direct greater attention to this population, as well as to conduct studies in order to help them achieve optimal levels of health and well-being^(8-9)^.

Studies on caregiver SC have been conducted in several fields and contexts^(16-19)^, with different types of caregivers^(20-23)^. Regarding intervention studies focused on the SC of caregivers of older adults with dementia, a scoping review that included seven intervention studies aimed to explore the potential health benefits of interventions developed to improve the SC of family caregivers of people with dementia^(24)^. The authors concluded that the interventions did not focus exclusively on the improvement and measurement of SC, but were related to the promotion of health and healthy lifestyle, such as physical exercise. Recently, a meta-analysis of 15 studies with intervention to promote SC behavior among ICs of older patients with and without dementia presented as one of its conclusions that caregiver’s SC measures in research have been notably deficient^(25)^.

Self-efficacy (SE)—one of the pillars of the Social Cognitive Theory proposed by Albert Bandura in the 1980s—is defined as an individual’s belief in their own ability to perform actions necessary to achieve certain goals^(26)^. This belief influences a person’s behavior, thinking, feeling, and motivation, which evolves as they acquire new skills and experiences. People’s SE beliefs are formed through the interpretation of information from four main sources: domain experience, vicarious experience, social persuasion, and physiological and affective states^(27)^. These beliefs can impact the intensity of people’s commitment to perform a certain action, influencing their decisions and modifying how they cope with difficulties experienced^(28)^.

SE is an important determinant of SC behavior and can influence the decision to take care of oneself^(29)^. Higher self-efficacy may be related to better performance in self-care behavior^(29)^, that is, the higher an individual’s self-efficacy, the greater the effort and commitment to achieve their goals and to self-care.

There is limited evidence on interventions oriented toward improving the SC capacity of caregivers of older adults with dementia using SE as a conceptual basis. Only one study conducted an intervention to improve the SC of caregivers of older adults with dementia, using this construct^(30)^. The researchers evaluated a telephone-based exercise guidance intervention programmed to improve SE for SC in 137 women caring for spouses with dementia. The intervention group showed a significant increase in weekly physical exercise and a reduction in perceived stress after six months of follow-up compared to the control group. Self-efficacy for physical exercise was also significantly higher in the intervention group after 6 and 12 months of intervention^(30)^.

Considering the scarcity of intervention studies to improve the SC of caregivers of older adults with dementia using SE, the following question was asked: What is the effectiveness of an intervention oriented toward SC in family caregivers of older adults with dementia, when using SE beliefs as a basis for this intervention?

Thus, the objective of this study was to assess the effectiveness of a self-efficacy-based educational intervention to increase the self-care capacity of IC of older adults with dementia.

## Method

### Study type

This is a quasi-experimental quantitative study, with pre- and post-intervention assessment, without a control group.

### Study location

It was conducted at the geriatric outpatient clinic of the Júlio Muller University Hospital (HUJM), located in the city of Cuiabá/MT, Brazil.

### Study period

The study was conducted from August to December 2022.

### Population

The study population consisted of ICs of older adults with dementia treated at the geriatric outpatient clinic of HUJM.

### Sample

To determine the sample, we identified 838 medical consultations, in which 61 older adults were diagnosed with dementia. To identify the main caregiver, telephone contact was made with the family. In this contact, it was found that eight older adults had died; twenty-five telephone numbers were non-existent or impossible to receive calls; and in eight calls, after five attempts on alternate days, people did not answer. At the end, 20 ICs of older adults with dementia were eligible for the research.

### Inclusion criteria

The inclusion criteria for the research were: having been the main IC of the older adult with dementia for at least one year; being aged 18 years or older; and having access to a smartphone and the internet and knowing how to use the WhatsApp messaging app*.* We adopted the following exclusion criteria: presenting difficulty in communicating, hearing, and understanding the questions asked by the researcher.

### Discontinuity criteria

The discontinuity criteria were: not accessing the material sent after five attempts of feedback; not responding to messages or phone calls; and death of the older adult.

All 20 caregivers met the inclusion criteria and initiated the intervention protocol. Three caregivers were discontinued from the intervention protocol: two for not responding to messages and not answering phone calls, and one due to the death of the older adult. At the end, 17 ICs of older adults diagnosed with dementia completed the intervention protocol.

### Study endpoints and instruments

The primary endpoint of the study was the SC capacity of ICs of older adults with dementia. This endpoint was measured using the Appraisal of Self-care Agency Scale (ASA-A), translated into Portuguese as the *Escala para Avaliar a Capacidade de Autocuidado* (EACAC), with cross-cultural adaptation and validation^(31)^. The EACAC consists of 24 items, with five response options. The scale classifications are: very poor (24 to 40 points); poor (40 to 56 points); fair (56 to 72 points); good (72 to 88 points); very good (88 to 104 points); excellent (104 to 120 points)^(31)^.

The secondary endpoints were sociodemographic (sex; date of birth; age; marital status; relationship with the older adult; years of education; occupational status; monthly income; income source; and cohabitation with the older adult); health conditions (self-assessed current health status; smoking habit; alcohol use; health problem, how many health problems; what health problem; regular use and amount of medication); and caregiver care characteristics (time of care for the older adult with dementia – years, days of the week, and hours per day; previous experience as a caregiver; help to care for the older adult; type and frequency of help; level of overload and perceived SE). The Zarit Scale, translated and validated for Brazil^(32)^, was used to assess overload. The instrument has 22 items, scored from 0 to 4 points. The scale’s total score is obtained by adding all items and can range from 0 to 88 points^(32)^. This study adopted the following classification: without overload (<46 points), slight overload (46–56 points), and intense overload (>56 points)^(33)^. Perceived SE was measured using The General Self-efficacy Scale*,* adapted and validated for Brazil^(34)^, called the *Escala de Autoeficácia Geral Percebida* (EAEGP). The instrument has ten items, assessed using a 5-point Likert-like scale. In a range from 10 to 50 points, the higher the score, the higher the perceived SE^(34)^.

### Data collection

Data collection was conducted from August to December 2022, and sociodemographic, caregiver health condition and care characteristics, and SC capacity data were collected using the telephone interview technique^(35)^. Previously, a pilot test was carried out in the community with three ICs of older adults with dementia, with characteristics similar to those to be investigated. The technique and instruments were considered adequate for data collection.

**Figure 1-:**
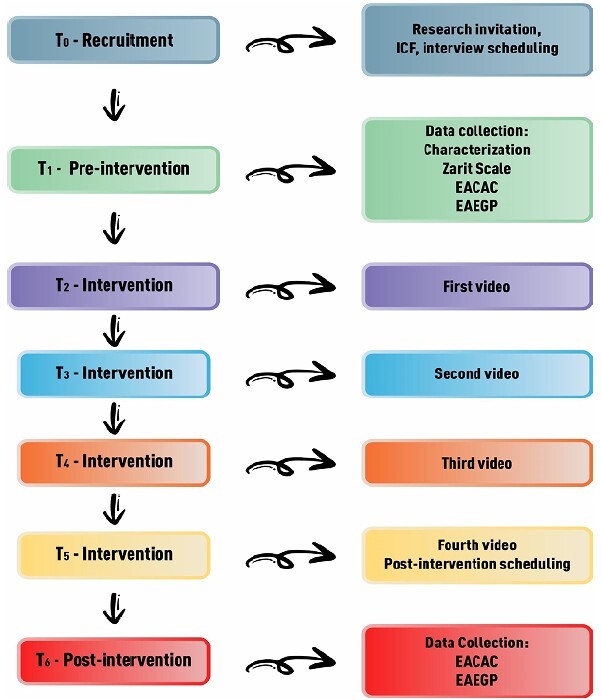
Times and activities of the educational intervention. Cuiabá, MT, Brazil, 2023

The educational intervention was carried out in the second half of 2022 in seven times (T_0_–T_6_) (Figure 1):

T_0_ – Recruitment: through telephone contact, the researcher informed the IC about the research, its procedures and purpose and invited them to participate in the study. In case of acceptance, the informed consent form (ICF) was read and recorded for caregivers who verbalized their agreement. Each caregiver received, via a messaging application, a version of the ICF containing the researcher’s signature. Subsequently, the best date and time for the interview was scheduled with them.

T_1_ – Pre-intervention: on the scheduled days and times, via telephone call, the researcher collected from each caregiver the data on sociodemographic aspects, health condition, care provided to the older adult, and applied the scales for overload (Zarit Scale), self-care capacity (EACAC), and self-efficacy (EAEGP). At the end, the caregivers were informed about the procedures of the next activities of the educational intervention.

T_2_ – Intervention: for the intervention, four videos were used as educational technology. These videos were produced by the researcher on the Vyond platform, with preparation of a storyboard, containing the illustrations/images that were used in the construction of the videos, following recommendations by Fleming, Reynolds, and Wallace^(36)^.

Likewise, the videos were validated as to content and appearance by expert judges in the health care field and in the field of technology and communication, respectively. The content of the videos had, as theoretical basis, the four SE belief sources of Albert Bandura’s Social Cognitive Theory^(37)^ (mastery experience, vicarious experience, social persuasion, and physiological and affective states) and a broad review of the literature on the SC of IC of older adults with dementia.

The first video lasted three minutes and forty-eight seconds and was based on the first source of SE belief—domain experience. The setting was a meeting of a support group for caregivers of older adults with dementia. The theme was the concept of self-care, some types of SC and possible means of practicing them. The objective was to encourage ICs to reflect on their SC experiences as caregivers of older adults with dementia. At the end, the nurse encouraged reflections on the topic and announced the content of the next meeting.

The video was sent by the WhatsApp messaging app to each IC. They were informed that they had five days to watch the video and that they could inform the researcher, at any time, about problems with the videos, as well as clarify doubts and make comments on the content watched. After five days, the researcher contacted each caregiver, through the messaging app, to ask about access to the material, if they had watched and understood the content. If they answered positively, the researcher asked them about the content of the video to confirm the information. After that, the second video was sent.

T_3_ – Intervention: based on the second source of SE belief—vicarious experience. The objective of this video was to encourage the IC, through observation of the experience of other caregivers of older adults with dementia, to reflect on the capacity to take care of themselves and help them realize that they can benefit from models that contribute to improving their form of self-care. To this end, using the same setting as the previous video, the nurse presented to the ICs the testimony of three caregivers about their self-care experiences and the benefits of this practice for quality of life and health. The duration of this video was seven minutes and fifty-seven seconds, and the theme was SC experiences of caregivers and the benefits of this practice for quality of life and health. As previously, five days later, the researcher repeated the aforementioned procedures and sent the third video.

T_4_ – Intervention: the third video was based on the third source of SE belief—social persuasion. The objective was to encourage and stimulate ICs to face the difficulties in performing self-care. The theme was scientific evidence on the benefits of SC that ICs of older adults living with dementia had through their practice. In this video, with duration of four minutes and eleven seconds, based on scientific evidence, the nurse addressed the benefits that the IC of older adults living with dementia had through the practice of self-care. Five days later, the researcher repeated the previous procedures and sent the fourth video.

T_5_ – Intervention: based on the fourth source of SE belief—physiological and affective states. This video aimed to inform the ICs on some strategies for coping with the difficulties experienced in performing self-care, and the theme was strategies for performing SC by the IC. The duration of the video was four minutes and fifty-one seconds, and in it the nurse exemplified some strategies for self-care, considering the difficulties reported by the caregivers. The video ended with the nurse saying goodbye to the ICs, making herself available to clarify their doubts. Five days later, after the previous procedures, the researcher informed that this stage was completed and scheduled with each IC the day and time to hold the post-intervention assessment.

T_6_ – Post-intervention: the researcher performed, via telephone call, a new assessment of SC capacity, applying the EACAC.

### Data analysis

Data analysis used the STATA program, version 16.1. Descriptive analysis was presented in tables and graphs, with absolute and relative frequency. To compare the data before and after the intervention, we performed the data normality test and the graphical presentation with boxplot. Subsequently, we performed a non-parametric Wilcoxon Rank-Sum test and a paired t-test and calculation of mean, standard deviation, median, and minimum and maximum. A significance level of 0.05 was considered.

## Ethical aspects

The research project was approved under opinion 4,622,301 by the Research Ethics Committee of the Júlio Müller University Hospital and registered in the Brazilian Registry of Clinical Trials (ReBEC) under number RBR-7gfvn3y.

## Results

Of the 17 ICs of older adults with dementia in the research, the majority are female (88.2%) and have more than 11 years of education (58.8%), 41.2% are in the 50–59 years age group, and 47% are married or in a stable union. Regarding occupation, 35.3% do not work. As for income, 29.4% earn up to one monthly minimum wage and 29.4% have no income. Almost half of the income of the ICs (41.7%) comes from retirement, and 41.7% comes from work. Most caregivers are the son/daughter of the older adults (76.5%), 88.2% live with them, 53.3% for 15 years or more.

With regard to health conditions, 41.2% of the ICs self-assess their health as regular. The majority (88.2%) do not smoke, and 64.7% do not use alcohol. Regarding health problems, only 23.6% claim to have three or more, and the main problems reported are cardiovascular (40.0%), psychiatric (40.0%), and endocrine (30.0%). Most (52.9%) reported that they do not use medications, and 29.4% use two to four medications regularly.

Regarding activities as a caregiver, 29.4% have cared for the older adult for a period of 5 to 9 years, and 29.4% have cared for 10 years or more. Most report no previous experience as a caregiver (52.9%). Most report providing care every day of the week (76.4%), most for 24 hours (88.2%). The highest number of ICs (76.5%) claim to receive some type of help with the older adult, and 53.8% report that the type of help they receive the most is in the care of the older adult. Regarding the level of overload, assessed using the Zarit Scale, most caregivers (70.6%) were classified in the category without overload. The majority (94.1%) had a good score in the measurement of perceived SE evaluated by the EAEGP.

**Figure 2-:**
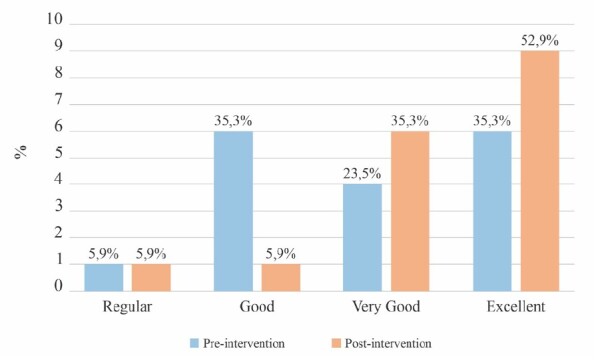
Comparison of self-care capacity before and after the educational intervention. Cuiabá, MT, Brazil, 2023

Figure 2 shows the pre- and post-intervention assessment of SC capacity.

**Figure 3-:**
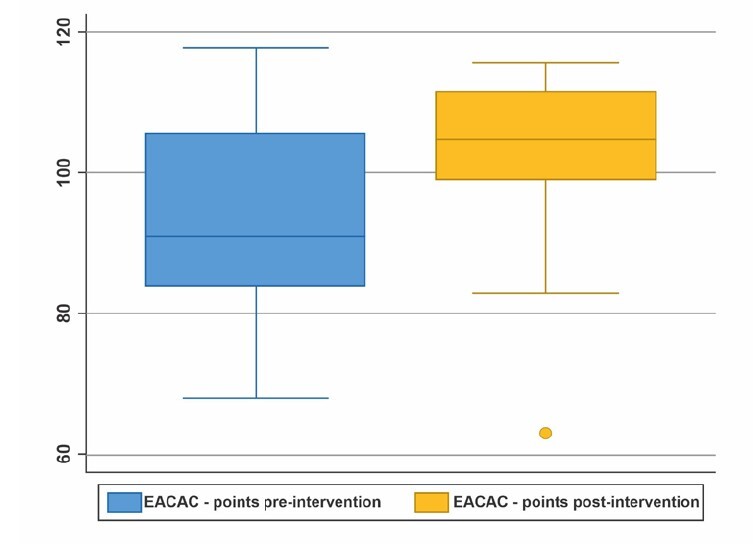
Boxplot of the comparison analysis of self-care capacity pre and post intervention. Cuiabá, MT, Brazil, 2023

Table 1 and Figure 3 present the comparison analysis of the effect of the educational intervention on the SC capacity of ICs. Table 1 shows the pre- and post-intervention values for the mean, median, standard deviation, minimum and maximum, and the p-value.

**Table 1-:** Comparison analysis of the effect pre and post educational intervention. Cuiabá, MT, Brazil, 2023

**Assessment**	**Mean**	**Median**	*SD	**Min./Max.** ^†^	p **-value**
**Self-care capacity**					**0.029** ^‡^
Pre	95.00	091.00	14.40	68;118	
Post	102.11	105.00	13.39	63;116	

*SD = Standard Deviation; ^†^Min./Max. = Minimum and maximum; ^‡^Wilcoxon paired test

The boxplot (Figure 3) shows the difference of the medians, the dispersion of the data and the differences between the 3rd and 1st quartiles in the comparison pre and post intervention.

*EACAC = *Escala para Avaliar a Capacidade de Autocuidado*

**Figure 4-:**
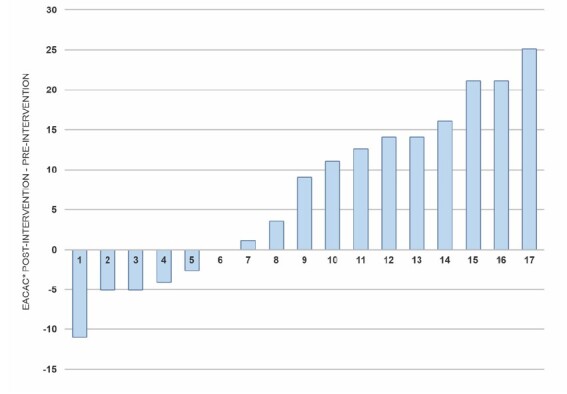
Intra-individual comparison of the scores of the *Escala para Avaliar a Capacidade de Autocuidado* post educational intervention. Cuiabá, MT, Brazil, 2023

Based on the intra-individual comparative analysis of SC capacity, Figure 4 shows the scores in the comparison pre and post intervention.

*EACAC = *Escala para Avaliar a Capacidade de Autocuidado*

## Discussion

The main finding of this study shows that the intervention was effective in increasing the SC capacity of ICs, especially in relation to the change in the scores of those who were in the good category.

A point to be considered in the analysis is that most of the participants in the sample of this study have conditions that may contribute to SC. Although they have cared for the older adults for a long period of time, providing care every day of the week and for 24 hours, they were classified as without overload. Perhaps because most receive some kind of help with the older adult, both in care and in financial terms. The literature shows that under these conditions SC can be better practiced, as in the study on factors related to overload and SC for hypertension in 68 family caregivers of older adults, which showed that caregivers assisted by another person in the care of the older adult had a higher score in the domain of confidence level regarding their hypertensive condition, when compared to those who did not receive assistance. The longer the time of care provided, the lower the score of the domain of SC management measures during pressure decompensation^(38)^.

Nevertheless, it should be considered that the participants of this study, after the intervention, started to present better scores for SC capacity, increasing the percentage values of the very good and excellent categories. Probably, this result occurred because they also had a good SE score before the intervention.

The literature shows that SE is a determinant of SC behavior and that it can influence the decision to take care of oneself^(29)^. Higher SE scores are associated with improved general health, quality of life, mental health and self-esteem, social functioning, and SC capacity with assertive decision-making^(39)^. Additionally, higher SE may be related to better performance in SC behavior^(40)^, that is, the higher the SE of an individual, the greater the effort and commitment to achieve their goals and to self-care^(41)^. SE beliefs impact the intensity of people’s commitment to perform a certain action, and can influence their decisions and modify how they cope with the difficulties experienced^(28)^.

Furthermore, the SE-based intervention may have contributed to further increase the SC capacity of the ICs. The fact that the intervention worked on the SE sources in order to influence the increase in SC may have improved the SE of these caregivers, which, in turn, contributed to the increase in SC. Seemingly, encouraging the individual to think about their SC experiences, reflect on the benefits of SC for their life, share the positive SC experiences of other caregivers, and encourage them to adopt SC measures influenced the SC behavior of the caregivers in this study. This was also found in studies that used the SE construct in educational interventions to improve the SC behavior of patients submitted to radical prostatectomy^(42)^, hemodialysis patients^(43)^, and pregnant women^(44)^.

Similarly, it can be considered that the level of education of the caregivers in this research favored the increase in the SC capacity of these caregivers, since most had 11 years or more of education. The literature has proven the association between the level of education and SC behaviors^(45-47)^, demonstrating that it can be a conditioning factor in how individuals engage and perform SC actions, as it reflects their capacity to understand the contents and guidelines contained in educational interventions^(46,48)^.

The comparative analysis of SC capacity presented in the boxplot reaffirms that there was an increase in the caregivers’ SC capacity and effectiveness of the educational intervention, as there was a significant change in score distribution and data dispersion before and after the intervention.

However, although the results are positive, it should be considered that, in the intra-individual comparative analysis of SC capacity, there was a maintenance and decrease in the score of the responses of some research participants after the educational intervention. This may be due to the intervention being based on SE sources. Each source has the objective of motivating the individual to reflect on their SE capacity to perform certain behaviors. According to Bandura^(37)^, individuals have the capacity to reflect on the value and meaning of their actions and, if necessary, make adjustments. Each individual, based on the same experience, cognitively processes the sources of SE, and each interpretation can lead to different efficacy beliefs^(49)^.

SE belief sources can influence the individual’s perception of their actions and behaviors. If these SE sources indicate that their actions/behaviors are in line with expectations, the person may perceive that they are performing an appropriate behavior. However, if they indicate difficulties, failures, or inadequacy, the person may perceive the need to adjust it^(27)^.

Thus, it is possible that these caregivers, when exposed to the situations worked on in each video, had the opportunity to reflect on their SC and that of other people. For example, when the SE sources “Domain Experience” and “Vicarious Experience” were worked on in the videos to encourage caregivers to reflect on their SC experiences, maybe those whose scores decreased after the intervention reconsidered their SC practices. The intervention may have influenced the perceived SE (perception of one’s own capacity) of the ICs and motivated them to adjust their behavior, reaching the conclusion that what they practiced was not SC or that their SC was insufficient.

This study has some limitations. The sample size reduces the possibility of generalizing the results and their applicability to other situations. In addition, with a small sample, variability in results can be high. However, the statistical tests proved that, in this population, this variability did not occur; on the contrary, there was a decrease in data dispersion. This improves the internal validity of the study, thus suggesting that the differences observed before and after may be because of the intervention and not other factors. Otherwise, less variability means that the results are more reliable.

The results of this study represent an advance in scientific knowledge about the care that ICs provide for older adults, specifically to those living with dementia. SC is a fundamental element in this care, and educational interventions that contribute to its occurrence are very important, especially for nurses who have the opportunity to work closely with this population. Therefore, there is potential for nursing to educate ICs, contributing to the development of SC and mitigating the overload related to the care of older adults with dementia with benefits for their quality of health and life.

Further intervention studies on the SC of ICs of older adults with dementia is recommended, with larger samples that enable the generalization of the results. Moreover, similar studies, but longitudinal, are suggested, in order to monitor and provide results over time, in addition to research with a randomized clinical trial design, with larger samples.

## Conclusion

The SE-based educational intervention was effective in increasing the SC capacity of ICs of older adults living with dementia, since it obtained statistically significant post-intervention values compared to pre-intervention values. This result was probably due to the educational intervention having adopted videos based on the SE belief sources in order to influence the increase in SC. Furthermore, contributing factors may include the participants having good level of education, receiving some type of help in caring for the older adults, having no overload, and already having a good SE score before the intervention.

## Data Availability

All data generated or analysed during this study are included in this published article.
