# Supplementary material for: Self-care capacity of informal caregivers of older adults with dementia: quasi-experimental study
Source: Rev Lat Am Enfermagem. 2025 Oct 27;33:e4677. doi: 10.1590/1518-8345.7715.4677 (PMC12562611; doi:10.1590/1518-8345.7715.4677)
Supplement: Translation

# Translation: Capacidade de autocuidado de cuidadores informais de pessoas idosas com demência: estudo quase experimental*

**DOI:** 10.1590/1518-8345.7715.4678

**Authors:** Daniela Luzia Zagoto Agulho, Annelita Almeida Oliveira Reiners, Rosemeiry Capriata de Souza Azevedo, Adriana Delmondes de Oliveira, Carla Rafaela Teixeira Cunha, Amanda Cristina de Souza Andrade, Joana Darc Chaves Cardoso, Tiago Reboulas Mazza

**Affiliations:** 1 Universidade Federal de Mato Grosso, Faculdade de Enfermagem, Cuiabá, MT, Brasil; 2 Faculdade Fasipe Cuiabá, Departamento de Enfermagem, Cuiabá, MT, Brasil

**Keywords:** Cuidadores, Demência, Autocuidado, Autoeficácia, Educação, Educação em Enfermagem

## Abstract

avaliar a efetividade de uma intervenção educativa baseada na autoeficácia para aumentar a capacidade de autocuidado de cuidadores informais de pessoas idosas com demência.

estudo quase experimental, com avaliação antes e depois, sem grupo controle. Participaram 17 cuidadores informais de pessoas idosas com demência. A intervenção foi realizada em três etapas: Pré-intervenção: coleta de dados para caracterização dos cuidadores, medidas de sobrecarga, capacidade de autocuidado e autoeficácia percebida; Intervenção: realizada por meio de quatro vídeos produzidos pela própria pesquisadora com base nas quatro fontes de crença de autoeficácia da Teoria Social Cognitiva de Bandura; Pós-intervenção: nova medida de capacidade de autocuidado. Foram realizadas análises descritivas, teste de normalidade e teste não paramétrico de Wilcoxon *Rank-Sum* e teste t pareado, considerando nível de significância de 0,05. A pesquisa teve aprovação pelo Comitê de Ética em Pesquisa, sob o parecer 4.622.301, e foi registrada no Registro Brasileiro de Ensaios Clínicos – RBR-7gfvn3y.

na análise de comparação do efeito da intervenção educativa sobre a capacidade de autocuidado, constatou-se aumento no valor da média (102,11) na pós-intervenção em relação ao valor da média da pré-intervenção (95,00), com significância estatística (p=0,029).

a intervenção educativa baseada na autoeficácia foi efetiva no aumento da capacidade de autocuidado dos cuidadores informais de idosos com demência.

## Destaques

**(1)** A produção de vídeos educativos é válida e efetiva em intervenções da enfermagem. **(2)** A autoeficácia pode melhorar o autocuidado de cuidadores de idosos com demência. **(3)** A intervenção com vídeos foi efetiva na melhora do autocuidado dos cuidadores. **(4)** Intervenções da enfermagem podem promover o autocuidado de cuidadores de idosos.

## Introdução

A *World Health Organization* (WHO) estimou que, em 2021, aproximadamente 55 milhões de pessoas em todo o mundo apresentavam demência, e projeções indicam que esse número, em 2050, pode ultrapassar 130 milhões^(1)^. Dos 10 milhões de novos casos registrados anualmente, seis milhões ocorrem em países de baixa e média renda^(2)^. No Brasil, estimava-se que, em 2019, 1,85 milhão de brasileiros viviam com doença de Alzheimer (DA) e outras formas de demência^(3)^. A DA é a mais prevalente, responsável por aproximadamente 70% dos casos^(4)^.

Por ser uma síndrome irreversível e progressiva, a demência acarreta prejuízos mentais, físicos e sociais aos seus portadores e requer, ao longo do tempo, assistência de um cuidador informal (CI), uma pessoa, geralmente um familiar, que, motivada por vínculos afetivos, emocionais ou parentais, responsabiliza-se total ou parcialmente pelo cuidado não remunerado de um familiar idoso dependente^(5)^.

No Brasil, ainda não existem dados sociodemográficos específicos sobre as pessoas que desempenham o papel de CI de pessoas idosas acometidas pela demência. Sabe-se apenas que é crescente o número de familiares que prestam o cuidado a pessoas idosas no país, pois, de acordo com dados do censo de 2020^(6)^, a quantidade de familiares que desempenhavam esse papel saltou de 3,7 milhões, em 2016, para 5,1 milhões, em 2019. Uma pesquisa realizada com cuidadores remunerados e não remunerados de diversos países, incluindo o Brasil^(7)^, mostrou que a maioria dos CI é composta por mulheres, de diferentes etnias e nível educacional secundário. Nos Estados Unidos, em 2023^(8)^, mais de 11 milhões de pessoas cuidavam de pessoas idosas acometidas pelo Alzheimer e outras formas de demência.

Comumente, o cuidado de pessoas com demência acarreta impacto negativo na vida dos CI^(9)^, podendo afetar sua saúde física, psicológica, emocional e social, além de gerar sobrecarga^(8-9)^, que pode comprometer a qualidade do cuidado prestado à pessoa idosa e o cuidado que o cuidador dedica a si mesmo, isto é, o autocuidado (AC)^(10)^.

A WHO^(11)^ define o AC como a habilidade de as pessoas promoverem sua saúde e prevenirem doenças ou incapacidades. O AC é considerado o fator principal para o bem-estar físico e mental das pessoas^(12)^. Trata-se de uma prática universal necessária para manter relações saudáveis consigo e com os outros, contribuindo diretamente para o bem-estar geral e a prevenção de doenças ou complicações, abrangendo aspectos como saúde, pensamentos, emoções e atitudes^(13)^. Ao dedicar tempo e atenção aos cuidados pessoais, elas conseguem fortalecer sua capacidade de lidar com o estresse e as demandas do cotidiano e até mesmo com a sobrecarga^(14)^. No caso dos cuidadores, o AC, além de favorecer o bem-estar, possibilita maior qualidade na assistência prestada às pessoas idosas com demência^(15)^. Nesse sentido, há recomendações sobre a necessidade de se direcionar maior atenção a essa população, bem como de desenvolver estudos a fim de ajudá-la a atingir níveis ideais de saúde e bem-estar^(8-9)^.

Estudos sobre o AC de cuidadores têm sido conduzidos em diversas áreas e contextos^(16-19)^, com diferentes tipos de cuidadores^(20-23)^. No que se refere a estudos de intervenção voltados para o AC de cuidadores de pessoas idosas com demência, uma revisão de escopo que incluiu sete estudos de intervenção objetivou explorar os potenciais benefícios de saúde das intervenções desenvolvidas para melhorar o AC de cuidadores familiares de pessoas com demência^(24)^. Os autores concluíram que as intervenções não tiveram foco exclusivo na melhora e medida do AC, mas estavam ligadas à promoção da saúde e ao estilo de vida saudável, como exercícios físicos. Recentemente, uma meta-análise de 15 estudos de intervenção para promover o comportamento de AC entre CI de pacientes idosos com e sem demência apresentou como uma de suas conclusões que as medidas do AC dos cuidadores nas pesquisas tem sido notavelmente deficientes^(25)^.

A autoeficácia (AE), um dos pilares da Teoria Social Cognitiva proposta por Albert Bandura na década de 80, é definida como a crença do indivíduo em sua própria capacidade de realizar ações necessárias para atingir determinados objetivos^(26)^. Essa crença influencia comportamento, pensamento, sentimento e motivação da pessoa, que evoluem conforme ela adquire novas habilidades e experiências. As crenças de AE das pessoas são formadas a partir da interpretação de informações advindas de quatro fontes principais: experiência de domínio, experiência vicária, persuasões sociais e estados somáticos e emocionais^(27)^. Essas crenças podem auxiliar na intensidade de empenho que as pessoas colocam para realizar uma determinada ação, influenciando suas decisões e modificando o enfrentamento das dificuldades vivenciadas^(28)^.

A AE é um determinante importante do comportamento de AC e pode influenciar a decisão de cuidar de si^(29)^. Maior autoeficácia pode estar relacionada com melhor desempenho no comportamento de autocuidado^(29)^, ou seja, quanto maior a autoeficácia de um indivíduo, maior será o esforço e empenho para alcançar seus objetivos e para se autocuidar.

Há evidências limitadas sobre intervenções voltadas para a melhora da capacidade do AC de cuidadores de idosos com demência utilizando a AE como base conceitual. Apenas um estudo realizou uma intervenção para melhorar o AC de cuidadores de pessoas idosas com demência, utilizando esse construto^(30)^. Os pesquisadores avaliaram uma intervenção de orientação para a realização de exercícios físicos por telefone programada para melhorar a AE para o AC de 137 mulheres cuidadoras de cônjuges com demência. O grupo intervenção demonstrou aumento significativo na prática de exercícios físicos semanais e redução no estresse percebido após seis meses de acompanhamento em relação ao grupo controle. A autoeficácia para a realização de exercícios físicos também foi significativamente maior no grupo intervenção, após 6 e 12 meses da intervenção^(30)^.

Considerando a escassez de estudos de intervenção para melhorar o AC de cuidadores de pessoas idosas com demência utilizando a AE, questionou-se: Qual a efetividade de uma intervenção voltada para o AC em cuidadores familiares de idosos com demência, quando se utiliza as crenças de AE como base para essa intervenção?

Desse modo, o objetivo deste estudo foi avaliar a efetividade de uma intervenção educativa baseada na autoeficácia para aumentar a capacidade de autocuidado de CI de pessoas idosas com demência.

## Método

### Tipo de estudo

Trata-se de estudo de abordagem quantitativa do tipo quase experimental, com avaliação antes e depois, sem grupo controle.

### Local do estudo

Foi realizado no ambulatório de geriatria do Hospital Universitário Júlio Muller (HUJM), localizado no município de Cuiabá/MT, Brasil.

### Período do estudo

O estudo foi realizado no período de agosto a dezembro de 2022.

### População

A população do estudo foi composta por CI de pessoas idosas com demência atendidos no ambulatório de geriatria do HUJM.

### Amostra

Para a determinação da amostra, foram identificadas 838 consultas médicas, nas quais verificou-se 61 pessoas idosas com diagnóstico de demência. Para a identificação do cuidador principal, foi realizado contato telefônico com a família. Nesse contato, observou-se que oito pessoas idosas haviam falecido; vinte e cinco números de telefones eram inexistentes ou impossibilitados de receber chamadas; e em oito ligações, após cinco tentativas em dias alternados, as pessoas não atenderam. Ao final, 20 CI de pessoas idosas com demência foram elegíveis para a pesquisa.

### Critérios de seleção

Os critérios de inclusão para a pesquisa foram: ser o CI principal da pessoa idosa com demência há, no mínimo, um ano; ter idade igual ou superior a 18 anos; e ter acesso a um smartphone e à internet e saber usar o aplicativo de mensagem *WhatsApp.* Como critério de exclusão: apresentar dificuldade de comunicação, de audição e de compreensão das perguntas realizadas pela pesquisadora.

### Critério de descontinuidade

Os critérios de descontinuidade foram: não acessar o material enviado, após cinco tentativas de feedback; não responder às mensagens ou aos telefonemas; e falecimento da pessoa idosa.

Todos os 20 cuidadores atenderam aos critérios de inclusão e iniciaram o protocolo de intervenção. Três cuidadores foram descontinuados do protocolo de intervenção, dos quais, dois por não responderem às mensagens e não atenderem aos telefonemas, e um devido ao falecimento do idoso. Ao final, 17 CI de pessoas idosas com diagnóstico de demência concluíram o protocolo da intervenção.

### Desfechos do estudo e instrumentos

O desfecho primário do estudo foi a capacidade para o AC dos CI de pessoas idosas com demência. Esse desfecho foi medido por meio da *Appraisal of Self-care Agency Scale* (ASA-A), traduzida para o português como Escala para Avaliar a Capacidade de Autocuidado (EACAC), com adaptação transcultural e validação^(31)^. A EACAC é formada por 24 itens, com cinco opções de respostas. A classificação da escala é: péssima (24 a 40 pontos); ruim (40 a 56 pontos); regular (56 a 72 pontos); boa (72 a 88 pontos); muito boa (88 a 104 pontos); ótima (104 a 120 pontos)^(31)^.

Já os desfechos secundários foram os sociodemográficos (sexo; data de nascimento; idade; estado civil; relação com o idoso; anos de estudo; situação ocupacional; renda mensal; renda proveniente de; e se reside com o idoso); de condições de saúde (autoavaliação do estado de saúde atual; se fuma; uso de bebida alcoólica; problema de saúde, quantos problemas de saúde; qual problema de saúde; uso regular e quantidade de medicamento); e de características do cuidado do cuidador (tempo de cuidado da pessoa idosa com demência – anos, dias da semana e horas por dia; experiência anterior como cuidador; ajuda para cuidar da pessoa idosa; tipo e frequência da ajuda; nível de sobrecarga e AE percebida). Para a avaliação da sobrecarga, foi utilizada a Escala de Zarit, traduzida e validada para o Brasil^(32)^. O instrumento apresenta 22 itens, pontuados de 0 a 4 pontos. A pontuação total da escala é obtida somando todos os itens e pode variar de 0 a 88 pontos^(32)^. Neste estudo, foi utilizada a seguinte classificação - sem sobrecarga (< 46 pontos), sobrecarga ligeira (de 46 a 56 pontos) e sobrecarga intensa (> 56 pontos)^(33)^. A AE percebida foi verificada por meio da *The General Self-efficacy Scale,* adaptada e validada para o Brasil^(34)^, denominada Escala de Autoeficácia Geral Percebida (EAEGP). O instrumento conta com dez itens, avaliados por meio de uma escala do tipo Likert de 5 pontos. Em um intervalo de 10 a 50 pontos, quanto maior a pontuação, maior a percepção de AE^(34)^.

### Coleta de dados

A coleta de dados ocorreu no período de agosto a dezembro de 2022, e foram coletados os dados sociodemográficos, de condições de saúde e características do cuidado do cuidador e a capacidade de AC, utilizando-se a técnica de entrevista via telefone^(35)^. Antes, foi realizado um teste-piloto com três CI de pessoas idosas da comunidade com demência, com características semelhantes aos investigados. A técnica e os instrumentos foram considerados adequados para a coleta de dados.

A intervenção educativa foi realizada no segundo semestre de 2022 em sete tempos (T_0_ a T_6_) (Figura 1):

**Figura 1-:** Tempos e atividades da intervenção educativa. Cuiabá, MT, Brasil, 2023

T_0_ – Recrutamento: por meio de contato telefônico, a pesquisadora informou ao CI sobre a pesquisa, seus procedimentos e finalidade e convidou-o para a participação do estudo. Em caso de aceite, foram realizadas a leitura e a gravação do Termo de Consentimento Livre e Esclarecido (TCLE) ao cuidador que verbalizou sua concordância. Cada cuidador recebeu, via aplicativo de mensagem, uma versão do TCLE contendo a assinatura da pesquisadora. Na sequência, foi agendada com ele a melhor data e o horário para a entrevista.

T_1_ – Pré-intervenção: nos dias e horários agendados, via ligação telefônica, a pesquisadora coletou com cada cuidador os dados sociodemográficos, de condições de saúde, do cuidado prestado à pessoa idosa e aplicou as escalas de sobrecarga (Escala de Zarit), capacidade de autocuidado (EACAC) e autoeficácia (EAEGP). Ao final, os cuidadores foram informados sobre os procedimentos das próximas atividades da intervenção educativa.

T_2_ – Intervenção: para a realização da intervenção, foram utilizados quatro vídeos como tecnologia educativa. Esses vídeos foram produzidos pela própria pesquisadora, na plataforma *Vyond*, com elaboração de *storyboard*, contendo as ilustrações/imagens que foram utilizadas na construção dos vídeos, seguindo recomendações de Fleming, Reynolds e Wallace^(36)^.

Igualmente, os vídeos foram validados quanto ao conteúdo e à aparência por juízes especialistas da área da saúde e da área da tecnologia e comunicação, respectivamente. Para o conteúdo dos vídeos, utilizou-se como base teórica as quatro fontes de crenças da AE da Teoria Social Cognitiva de Albert Bandura^(37)^ (experiência de domínio, experiência vicária, persuasão verbal e estados somáticos e emocionais) e uma ampla revisão da literatura sobre o AC de CI de pessoas idosas com demência.

O primeiro vídeo teve duração de três minutos e quarenta e oito segundos e foi baseado na primeira fonte de crença da AE – experiência de domínio. O cenário foi de uma reunião de grupo de apoio a cuidadores de idosos com demência. O tema foi sobre o conceito de autocuidado, alguns tipos de AC e formas possíveis de praticá-los. O objetivo foi estimular os CI a refletir sobre suas experiências de AC como cuidador de pessoas idosas com demência. Ao final, a enfermeira incentivou as reflexões sobre o tema e anunciou o conteúdo do próximo encontro.

O vídeo foi enviado pelo aplicativo de mensagens *WhatsApp* a cada CI. Eles foram informados de que tinham cinco dias para assistir ao vídeo e que eles poderiam informar à pesquisadora, a qualquer momento, sobre problemas com os vídeos, bem como esclarecer dúvidas e fazer comentários sobre os conteúdos assistidos. Após cinco dias, a pesquisadora entrou em contato com cada cuidador, pelo aplicativo de mensagens, para perguntar sobre o acesso ao material, se haviam assistido e compreendido o conteúdo. Se respondessem de maneira afirmativa, a pesquisadora os questionava sobre o conteúdo do vídeo para confirmar a informação. Depois disso, o segundo vídeo foi enviado.

T_3_ – Intervenção: baseado na segunda fonte de crença da AE – experiência vicária –, o objetivo desse vídeo foi estimular o CI, por meio da observação da experiência de outros cuidadores de pessoas idosas com demência, a refletir sobre a capacidade de cuidar de si mesmo e ajudá-lo a perceber que pode se beneficiar de modelos que contribuem para melhorar a forma de autocuidado. Para isso, utilizando o mesmo cenário do vídeo anterior, a enfermeira apresentou aos CI o depoimento de três cuidadores sobre suas experiências de autocuidado e os benefícios dessa prática para a qualidade de vida e saúde. A duração desse vídeo foi de sete minutos e cinquenta e sete segundos, e o tema foi experiências de AC de cuidadores e os benefícios dessa prática para a qualidade de vida e saúde. Como procedeu anteriormente, cinco dias depois, a pesquisadora repetiu os procedimentos supracitados e enviou o terceiro vídeo.

T_4_ – Intervenção: o terceiro vídeo foi baseado na terceira fonte de crença de AE – persuasão verbal, e o objetivo foi encorajar e estimular os CI a enfrentar as dificuldades para realizar o autocuidado. O tema foi evidências científicas sobre os benefícios do AC que CI de pessoas idosas vivendo com demência tiveram por meio de sua prática. Nesse vídeo, com duração de quatro minutos e onze segundos, baseada em evidências científicas, a enfermeira abordou os benefícios que o CI de pessoas idosas vivendo com demência tiveram por meio da prática do autocuidado. Cinco dias depois, a pesquisadora repetiu os procedimentos anteriores e enviou o quarto vídeo.

T_5_ – Intervenção: baseado na quarta fonte de crença de AE – estados somáticos e emocionais, esse vídeo teve como objetivo possibilitar que os CI conhecessem algumas estratégias de enfrentamento das dificuldades vivenciadas para a realização do autocuidado, e o tema foi estratégias para realização do AC pelo CI. A duração do vídeo foi de quatro minutos e cinquenta e um segundos, e nele a enfermeira exemplificou algumas estratégias para o autocuidado, considerando as dificuldades relatadas pelos cuidadores. O vídeo foi finalizado com a enfermeira se despedindo dos CI, se colocando à disposição para sanar suas dúvidas. Cinco dias depois, após os procedimentos anteriores, a pesquisadora informou que essa etapa estava concluída e marcou com cada um dos CI o dia e horário para realizar a pós-intervenção.

T_6_ – Pós-intervenção: a pesquisadora realizou, via ligação telefônica, nova medida da capacidade de AC, aplicando a EACAC.

### Análise dos dados

A análise dos dados foi realizada com a utilização do programa STATA, versão 16.1. A análise descritiva foi apresentada em tabelas e gráficos, com frequência absoluta e relativa. Para comparação dos dados antes e após a intervenção, foram realizados o teste de normalidade dos dados e a apresentação gráfica com *boxplot*. Posteriormente, foi realizado um teste não paramétrico de Wilcoxon *Rank-Sum* e um teste t pareado e cálculo de média, desvio padrão, mediana, mínimo e máximo. Foi considerado um nível de significância de 0,05.

## Aspectos éticos

O projeto de pesquisa foi aprovado sob o parecer 4.622.301 pelo Comitê de Ética em Pesquisa do Hospital Universitário Júlio Müller e registrado no Registro Brasileiro de Ensaios Clínicos (ReBEC) sob o número RBR-7gfvn3y.

## Resultados

Dos 17 CI de pessoas idosas com demência pesquisados, a maioria é do sexo feminino (88,2%) e tem mais de 11 anos de estudo (58,8%), 41,2% estão na faixa etária de 50 a 59 anos e 47% são casados ou estão em uma união estável. Em relação à ocupação, 35,3% não trabalham. Quanto à renda, 29,4% recebem até um salário mínimo e outros 29,4% não a possuem. Quase metade da renda dos CI (41,7%) é proveniente de aposentadoria, e de 41,7% é do trabalho. A maioria dos cuidadores é filho/filha da pessoa idosa (76,5%), 88,2% reside com ela, 53,3% há 15 anos ou mais.

No que diz respeito às condições de saúde, 41,2% dos CI autoavaliam sua saúde como regular. A maioria (88,2%) não fuma, e 64,7% não fazem uso de bebida alcoólica. Sobre os problemas de saúde, apenas 23,6% afirmam ter três ou mais, e os principais problemas referidos são cardiovasculares (40,0%), psiquiátricos (40,0%) e endócrinos (30,0%). Grande parte (52,9%) refere não fazer uso de medicamentos, e 29,4% fazem uso de dois a quatro medicamentos regularmente.

No que se refere às atividades como cuidador, 29,4% deles estão cuidando da pessoa idosa por um período de 5 a 9 anos, e outros 29,4% cuidam por 10 anos ou mais. A maioria nega experiência anterior como cuidador (52,9%). Grande parte deles refere prestar o cuidado todos os dias da semana (76,4%), a maioria durante 24 horas (88,2%). O maior número de CI (76,5%) afirma receber algum tipo de ajuda com a pessoa idosa, e 53,8% referem que o tipo de ajuda que mais recebe é no cuidado com a pessoa idosa. Sobre o nível de sobrecarga, avaliado por meio da Escala de Zarit, a maioria dos cuidadores (70,6%) foi classificada na categoria sem sobrecarga. A maioria (94,1%) apresentou boa pontuação na medida de AE percebida avaliada pela EAEGP.

A avaliação da capacidade de AC na pré e pós-intervenção é apresentada na Figura 2.

**Figura 2-:** Comparação da capacidade de autocuidado antes e após a intervenção educativa. Cuiabá, MT, Brasil, 2023

A Tabela 1 e a Figura 3 apresentam a análise de comparação do efeito da intervenção educativa sobre a capacidade de AC dos CI. Na Tabela 1 constam os valores da pré e da pós-intervenção no que diz respeito à média, à mediana, ao desvio padrão, à mínima e à máxima e ao valor de *p*.

**Tabela 1-:** Análise de comparação do efeito da pré e pós-intervenção educativa. Cuiabá, MT, Brasil, 2023

**Variável**	**Média**	**Mediana**	DP*	**Mín./Máx.** ^†^	***p* valor**
**Capacidade de autocuidado**					**0,029** ^‡^
Pré	95,00	091,00	14,40	68;118	
Pós	102,11	105,00	13,39	63;116	

*DP = Desvio-Padrão; ^†^Mín./Máx. = Mínima e máxima; ^‡^Teste pareado de Wilcoxon

No *boxplot* (Figura 3) é possível verificar a diferença das medianas, a dispersão dos dados e as diferenças entre o 3º e o 1º quartis na comparação pré e pós-intervenção.

**Figura 3-:** *Boxplot* da análise comparativa da capacidade de autocuidado na pré e pós-intervenção. Cuiabá, MT, Brasil, 2023

*EACAC = Escala para Avaliar a Capacidade de Autocuidado

Com base na análise comparativa intraindividual da capacidade de AC, a Figura 4 mostra as pontuações na comparação antes e depois da intervenção.

**Figura 4-:** Comparação intraindividual das pontuações da Escala para Avaliar a Capacidade de Autocuidado após a intervenção educativa. Cuiabá, MT, Brasil, 2023

*EACAC = Escala para Avaliar a Capacidade de Autocuidado

## Discussão

O principal achado deste estudo mostra que a intervenção foi efetiva no aumento da capacidade de AC dos CI, principalmente em relação à mudança nos escores daqueles que se encontravam na categoria boa.

Um ponto a ser considerado na análise é que a maioria dos participantes da amostra deste estudo tem condições que podem contribuir para o AC. Embora cuidem das pessoas idosas por um período longo de tempo, prestando cuidado todos os dias da semana e durante 24 horas, foram classificados como não tendo sobrecarga. Talvez porque a maioria receba algum tipo de ajuda com a pessoa idosa, tanto no cuidado quanto no financeiro. A literatura evidencia que nessas condições o AC pode ser melhor praticado, como no estudo sobre os fatores relacionados à sobrecarga e ao AC para hipertensão em 68 cuidadores familiares de pessoas idosas, que evidenciou que cuidadores auxiliados por outra pessoa no cuidado com a pessoa idosa apresentaram maior escore no domínio grau de confiança referente à sua condição de hipertenso, quando comparados aos que não recebiam auxílio. Quanto maior o tempo de cuidado prestado, menor o escore do domínio medidas de manejo do AC durante a descompensação pressórica^(38)^.

Do mesmo modo, há que se considerar que os participantes deste estudo, após a intervenção, passaram a apresentar melhores escores na capacidade de AC, aumentando os valores percentuais das categorias muito boa e ótima. Provavelmente, esse resultado tenha ocorrido porque eles também já tinham uma boa pontuação da AE antes da intervenção.

A literatura mostra que a AE é um determinante do comportamento de AC e que pode influenciar a decisão de cuidar de si^(29)^. Maiores escores de AE estão associados com melhorias na saúde em geral, na qualidade de vida, na saúde mental e autoestima, no funcionamento social e na capacidade de AC com tomada de decisão assertiva^(39)^. Além disso, maior AE pode estar relacionada com melhor desempenho no comportamento de AC^(40)^, ou seja, quanto maior a AE de um indivíduo, maior será o esforço e empenho para alcançar seus objetivos e para se autocuidar^(41)^. As crenças de AE auxiliam na intensidade de empenho que as pessoas colocam para realizar uma determinada ação, podendo influenciar suas decisões e modificar o enfrentamento das dificuldades vivenciadas^(28)^.

Ademais, a intervenção baseada na AE pode ter contribuído para aumentar ainda mais a capacidade de AC dos CI. O fato de a intervenção ter trabalhado as fontes de AE no intuito de influenciar o aumento do AC, pode ter melhorado a AE desses cuidadores, o que, por sua vez, contribuiu para o aumento do AC. Parece que estimular o indivíduo a pensar sobre suas experiências de AC, a refletir sobre os benefícios do AC para a sua vida, compartilhar as experiências positivas de AC de outros cuidadores e encorajá-lo a adotar medidas de AC influenciou no comportamento de AC dos cuidadores deste estudo. Isso também foi encontrado em estudos que utilizaram o construto da AE nas intervenções educativas para melhorar o comportamento de AC de pacientes submetidos à prostatectomia radical^(42)^, pacientes em hemodiálise^(43)^ e mulheres grávidas^(44)^.

Da mesma forma, pode-se considerar que o nível de escolaridade dos cuidadores desta pesquisa tenha favorecido o aumento da capacidade de AC desses cuidadores, pois a maioria tinha 11 anos ou mais de estudo. A literatura tem comprovado a associação entre o nível de escolaridade e os comportamentos de AC^(45-47)^, demonstrando que ela pode ser um fator condicionante na forma como os indivíduos se envolvem e executam ações para o AC, pois reflete na sua capacidade de compreender os conteúdos e orientações contidas nas intervenções educativas^(46,48)^.

A análise comparativa da capacidade de AC apresentada no *boxplot* reafirma que houve aumento na capacidade de AC dos cuidadores e efetividade da intervenção educativa, pois nota-se que houve uma mudança significativa na distribuição dos escores e na dispersão dos dados antes e depois da intervenção.

Todavia, embora os resultados sejam positivos, há de se considerar que, na análise comparativa intraindividual da capacidade de AC, houve manutenção e diminuição na pontuação das respostas de alguns participantes da pesquisa após a intervenção educativa. Isso pode ter acontecido também em decorrência de a intervenção ter sido baseada nas fontes de AE. Cada fonte tem o objetivo de motivar o indivíduo a refletir sobre sua capacidade de AE para realizar determinados comportamentos. De acordo com Bandura^(37)^, o indivíduo tem a capacidade de refletir sobre o valor e o significado de suas ações e, se necessário, fazer ajustes. Cada indivíduo, a partir de uma mesma experiência, processa cognitivamente as fontes de AE, e cada interpretação pode levar a crenças de eficácia diferentes^(49)^.

As fontes de crenças de AE podem influenciar o indivíduo na percepção de suas ações e comportamentos. Se essas fontes de AE indicam que suas ações/comportamentos estão de acordo com o esperado, a pessoa pode perceber que está realizando um comportamento adequado. Mas se indicam dificuldades, falhas ou inadequação, a pessoa pode perceber a necessidade de ajustá-lo^(27)^.

Nesse sentido, é possível que esses cuidadores, ao serem expostos às situações trabalhadas em cada vídeo, tiveram a oportunidade de refletir sobre seu AC e o de outras pessoas. Por exemplo, quando as fontes de AE “Experiência de domínio” e “Experiência vicária” foram trabalhadas nos vídeos para estimular os cuidadores a refletir sobre suas experiências de AC, pode ser que aqueles cuja pontuação diminuiu após a intervenção reconsideraram suas práticas de AC. Talvez a intervenção tenha influenciado a AE percebida (percepção da própria capacidade) dos CI e os motivou a ajustar seu comportamento, chegando à conclusão de que o que praticavam não era AC ou que seu AC era insuficiente.

Este estudo apresenta algumas limitações. O tamanho da amostra reduz a possibilidade a generalização dos resultados obtidos e sua aplicabilidade para outras situações. Além disso, com uma amostra pequena, a variabilidade nos resultados pode ser alta. Porém, os testes estatísticos comprovaram que, nessa população, essa variabilidade não ocorreu, pelo contrário, houve uma diminuição da dispersão dos dados. Isso melhora a validade interna do estudo, de maneira a sugerir que as diferenças observadas antes e depois possam ser por causa da intervenção e não de outros fatores. De outro modo, menor variabilidade significa que os resultados são mais confiáveis.

Os resultados deste estudo representam um avanço no conhecimento científico sobre o cuidado que CI prestam às pessoas idosas, especificamente àquelas que vivem com demência. O AC é um elemento fundamental nesse cuidado, e intervenções educativas que contribuam para a sua ocorrência são muito importantes, principalmente para a enfermagem que tem oportunidade de trabalhar próximo a essa população. Há, portanto, potencial para a enfermagem realizar a educação de CI de modo a contribuir para o desenvolvimento do AC, mitigando a sobrecarga relacionada ao cuidado das pessoas idosas com demência com benefícios para sua qualidade de saúde e vida.

Recomenda-se que sejam realizados mais estudos de intervenção sobre o AC de CI de pessoas idosas com demência, aplicados em maiores amostras, que viabilizem a generalização dos resultados. Adicionalmente, sugere-se o desenvolvimento de estudos semelhantes, porém de natureza longitudinal, a fim de acompanhar e fornecer resultados ao longo do tempo, além de pesquisas com delineamento de ensaio clínico randomizado, com amostras maiores.

## Conclusão

A intervenção educativa baseada na AE foi efetiva no aumento da capacidade de AC de CI de pessoas idosas que vivem com demência, uma vez que obteve valores estatisticamente significativos na pós-intervenção em comparação à pré-intervenção. Esse resultado provavelmente ocorreu devido à intervenção educativa ter sido realizada por meio de vídeos baseados nas fontes de crenças de AE no intuito de influenciar o aumento do AC. Além disso, podem ter contribuído o bom nível de escolaridade dos participantes, o fato de receberem algum tipo de ajuda no cuidado à pessoa idosa, não terem sobrecarga e de já apresentarem uma boa pontuação da AE antes da intervenção.

## Data Availability

Todos os dados gerados ou analisados durante este estudo estão incluídos neste artigo publicado.

# Translation: Capacidad de autocuidado de cuidadores informales de personas mayores con demencia: un estudio cuasiexperimental*

**DOI:** 10.1590/1518-8345.7715.4676

**Authors:** Daniela Luzia Zagoto Agulho, Annelita Almeida Oliveira Reiners, Rosemeiry Capriata de Souza Azevedo, Adriana Delmondes de Oliveira, Carla Rafaela Teixeira Cunha, Amanda Cristina de Souza Andrade, Joana Darc Chaves Cardoso, Tiago Reboulas Mazza

**Affiliations:** 1 Universidade Federal de Mato Grosso, Faculdade de Enfermagem, Cuiabá, MT, Brasil; 2 Faculdade Fasipe Cuiabá, Departamento de Enfermagem, Cuiabá, MT, Brasil

**Keywords:** Cuidadores, Demencia, Cuidados personales, Autocuidado, Educación, Educación en Enfermería

## Abstract

evaluar la efectividad de una intervención educativa basada en la autoeficacia para aumentar la capacidad de autocuidado de cuidadores informales de personas mayores con demencia.

estudio cuasiexperimental, con evaluación antes y después, sin grupo de control. Participaron 17 cuidadores informales de personas mayores con demencia. La intervención se realizó en tres etapas: Preintervención: recopilación de datos para caracterizar a los cuidadores, medidas de sobrecarga, capacidad de autocuidado y autoeficacia percibida; Intervención: se realizó por medio de cuatro vídeos producidos por la propia investigadora basados en las cuatro fuentes de creencia de autoeficacia de la Teoría Social Cognitiva de Bandura; Posintervención: nueva medida de capacidad de autocuidado. Se realizaron análisis descriptivos, prueba de normalidad y prueba no paramétrica de Wilcoxon *Rank-Sum* y prueba t pareada, considerando un nivel de significancia de 0,05. La investigación fue aprobada por el Comité de Ética en Investigación, bajo el dictamen 4.622.301, y se registró en el Registro Brasileño de Ensayos Clínicos, con el número RBR-7gfvn3y.

en el análisis de comparación del efecto de la intervención educativa sobre la capacidad de autocuidado, se constató un aumento en el valor promedio (102,11) en la posintervención con relación al valor promedio de la preintervención (95,00), con significancia estadística (p=0,029).

la intervención educativa basada en la autoeficacia fue efectiva para aumentar la capacidad de autocuidado de cuidadores informales de personas mayores con demencia.

## Destacados

**(1)** La producción de vídeos educativos es válida y efectiva en las intervenciones de enfermería. **(2)** La autoeficacia puede mejorar el autocuidado de cuidadores de adultos mayores con demencia. **(3)** La intervención con vídeos fue efectiva para mejorar el autocuidado de los cuidadores. **(4)** Las intervenciones de enfermería pueden promover el autocuidado entre los cuidadores de personas mayores.

## Introducción

La *World Health Organization* (WHO) estimó que en el 2021, alrededor de 55 millones de personas en todo el mundo tenían demencia, y las proyecciones indican que este número, para el 2050, podría superar los 130 millones^(1)^. De los 10 millones de casos nuevos que se registran anualmente, seis millones se encuentran en países de ingresos bajos y medios^(2)^. En Brasil, se estimaba que, en el 2019, 1,85 millones de brasileños vivían con la enfermedad de Alzheimer (EA) y otras formas de demencia^(3)^. La EA es la más frecuente y es responsable de aproximadamente el 70% de los casos^(4)^.

Al ser un síndrome irreversible y progresivo, la demencia produce daños mentales, físicos y sociales a quienes la padecen y requiere, con el tiempo, la asistencia de un cuidador informal (CI), una persona, por lo general un familiar, que, motivada por vínculos afectivos, emocionales o parentales, asume total o parcialmente la responsabilidad del cuidado no remunerado de un familiar mayor dependiente^(5)^.

En Brasil, aún no existen datos sociodemográficos específicos sobre las personas que desempeñan el papel de CI de personas mayores afectadas por demencia. Solo se sabe que el número de familiares que brindan cuidados a personas mayores en el país está creciendo, ya que, según datos del censo del 2020^(6)^, el número de familiares que desempeñaron este papel aumentó de 3,7 millones en el 2016 a 5,1 millones en el 2019. Una encuesta realizada con cuidadores remunerados y no remunerados de varios países, incluido Brasil^(7)^, demostró que la mayoría de los CI son mujeres, de diferentes etnias y con nivel educativo secundario. En los Estados Unidos, en el 2023^(8)^, más de 11 millones de personas cuidaron a personas mayores que padecían Alzheimer y otras formas de demencia.

Comúnmente, el cuidado de personas con demencia acarrea un impacto negativo en la vida de los CI^(9)^ y puede afectar su salud física, psicológica, emocional y social, además de generar sobrecarga^(8-9)^, que puede comprometer la calidad del cuidado brindado a la persona mayor y el cuidado que el cuidador dedica a sí mismo, es decir, el autocuidado (AC)^(10)^.

La WHO^(11)^ define el AC como la habilidad de las personas para promover su salud y prevenir enfermedades o discapacidades. El AC se considera el factor principal para el bienestar físico y mental de las personas^(12)^. Se trata de una práctica universal necesaria para mantener relaciones saludables con uno mismo y con los demás, contribuyendo directamente al bienestar general y a la prevención de enfermedades o complicaciones, abarcando aspectos como la salud, los pensamientos, las emociones y las actitudes^(13)^. Al dedicar tiempo y atención a los cuidados personales, pueden fortalecer su capacidad para afrontar el estrés y las exigencias de la vida cotidiana y aún con la sobrecarga^(14)^. En el caso de los cuidadores, el AC, además de promover el bienestar, posibilita una mayor calidad en la atención brindada a las personas mayores con demencia^(15)^. En ese sentido, existen recomendaciones sobre la necesidad de dirigir mayor atención a esta población, así como desarrollar estudios con el fin de ayudarla a alcanzar niveles ideales de salud y bienestar^(8-9).^.

Se han realizado estudios sobre el AC de los cuidadores en diversas áreas y contextos^(16-19)^, con diferentes tipos de cuidadores^(20-23)^. En lo que se refiere a estudios de intervención centrados en el AC de los cuidadores de personas mayores con demencia, una revisión de alcance que incluyó siete estudios de intervención tuvo como objetivo explorar los posibles beneficios para la salud de las intervenciones desarrolladas para mejorar el AC de los cuidadores familiares de personas con demencia^(24)^. Los autores concluyeron que las intervenciones no se centraron exclusivamente en mejorar y medir el AC, sino que estaban vinculadas a la promoción de la salud y a un estilo de vida saludable, como la práctica de ejercicios físicos. Recientemente, un metaanálisis de 15 estudios de intervención para promover las conductas de AC entre los CI de pacientes mayores con y sin demencia presentó como una de sus conclusiones que las medidas de AC de los cuidadores en las investigaciones han sido notablemente deficientes^(25)^.

La autoeficacia (AE), uno de los pilares de la Teoría Social Cognitiva propuesta por Albert Bandura en la década de 1980, se define como la creencia del individuo en su propia capacidad para realizar acciones necesarias para alcanzar determinados objetivos^(26)^. Esta creencia influye en la actitud, pensamiento, sentimiento y motivación de una persona, que evolucionan a medida que adquiere nuevas habilidades y experiencias. Las creencias de AE de las personas se forman a partir de la interpretación de información procedente de cuatro fuentes principales: experiencia de dominio, experiencia vicaria, persuasiones sociales y estados somáticos y emocionales^(27)^. Estas creencias pueden ayudar en la intensidad del empeño que las personas ponen al llevar a cabo una determinada acción, influyendo en sus decisiones y modificando la forma de afrontar las dificultades que experimentan^(28)^.

La AE es un determinante importante de las conductas de AC y puede influir en la decisión de cuidarse a uno mismo^(29)^. Una mayor autoeficacia puede estar relacionada con un mejor desempeño en las conductas de autocuidado^(29)^, es decir, cuanto mayor sea la autoeficacia de un individuo, mayor será el esfuerzo y el empeño para lograr sus objetivos y para autocuidarse.

Existen evidencias limitadas sobre intervenciones dirigidas a mejorar la capacidad del AC de los cuidadores de personas mayores con demencia utilizando la AE como base conceptual. Solo un estudio realizó una intervención para mejorar el AC de cuidadores de personas mayores con demencia, utilizando este constructo^(30)^. Los investigadores evaluaron una intervención de orientación para realizar ejercicios físicos por teléfono diseñada para mejorar la AE para el AC de 137 mujeres cuidadoras de cónyuges con demencia. El grupo de intervención demostró un aumento significativo en la práctica de ejercicios físicos semanales y una reducción del estrés percibido después de seis meses de seguimiento en comparación con el grupo de control. La autoeficacia para realizar ejercicios físicos también fue significativamente mayor en el grupo de intervención, después de 6 y 12 meses de la intervención^(30)^.

Considerando la escasez de estudios de intervención para mejorar el AC de los cuidadores de personas mayores con demencia mediante AE, se planteó la pregunta: ¿Cuál es la efectividad de una intervención centrada en el AC de cuidadores familiares de personas mayores con demencia, cuando se utilizan las creencias de AE como base para esta intervención?

Así, el objetivo de este estudio fue evaluar la efectividad de una intervención educativa basada en la autoeficacia para incrementar la capacidad de autocuidado de CI de personas mayores con demencia.

## Método

### Tipo de estudio

Se trata de un estudio de enfoque cuantitativo de tipo cuasiexperimental, con evaluación antes y después, sin grupo control.

### Lugar de estudio

Se llevó a cabo en el ambulatorio geriátrico del Hospital Universitario Júlio Müller (HUJM), ubicado en el municipio de Cuiabá/MT, Brasil.

### Período de estudio

El estudio se llevó a cabo en el período de agosto a diciembre del 2022.

### Población

La población del estudio estuvo compuesta por CI de personas mayores con demencia atendidas en el servicio ambulatorio geriátrico del HUJM.

### Muestra

Para determinar la muestra, se identificaron 838 consultas médicas, en las cuales 61 adultos mayores fueron diagnosticados con demencia. Para identificar al cuidador principal, se realizó contacto telefónico con la familia. Durante este contacto, se observó que ocho personas mayores habían fallecido; veinticinco números de teléfono no existían o no podían recibir llamadas; y en ocho llamadas, después de cinco intentos en días alternos, las personas no atendieron. Finalmente, 20 CI de personas mayores con demencia fueron elegibles para la investigación.

### Criterios de selección

Los criterios de inclusión para la investigación fueron los siguientes: ser el CI principal de la persona mayor con demencia durante al menos un año; tener edad igual o superior a 18 años; y tener acceso a un teléfono inteligente y a internet y saber usar la aplicación de mensajería *WhatsApp.* Se excluyeron las personas que presentaban dificultades en la comunicación, audición y comprensión de las preguntas realizadas por la investigadora.

### Criterio de discontinuidad

Los criterios de discontinuidad fueron los siguientes: no acceder al material enviado, después de cinco intentos de *feedback*; no responder a los mensajes o a las llamadas telefónicas; y muerte de la persona mayor.

Los 20 cuidadores cumplieron con los criterios de inclusión y comenzaron el protocolo de intervención. Tres cuidadores fueron dados de baja del protocolo de intervención, dos de ellos por no responder a los mensajes ni atender a las llamadas telefónicas, y uno debido al fallecimiento de la persona mayor. Finalmente, 17 CI de personas mayores con diagnóstico de demencia completaron el protocolo de intervención.

### Desenlaces del estudio e instrumentos

El desenlace primario del estudio fue la capacidad de AC de los CI de personas mayores con demencia. Este desenlace se midió utilizando la *Appraisal of Self-care Agency Scale* (ASA-A), traducida al portugués como Escala para Evaluar la Capacidad de Autocuidado (EACAC), con adaptación transcultural y validación^(31)^. La EACAC consta de 24 ítems, con cinco opciones de respuesta. La clasificación de la escala es la siguiente: muy mala (24 a 40 puntos); mala (40 a 56 puntos); regular (56 a 72 puntos); buena (72 a 88 puntos); muy buena (88 a 104 puntos); excelente (104 a 120 puntos)^(31)^.

A su vez, los resultados secundarios fueron los sociodemográficos (sexo; fecha de nacimiento; edad; estado civil; relación con la persona mayor; años de estudio; situación laboral; ingresos mensuales; ingresos provenientes de; y si vive con la persona mayor); de condiciones de salud (autoevaluación del estado de salud actual; si fuma; si consume bebidas alcohólicas; si tiene problemas de salud, cuántos problemas de salud; qué problemas de salud; si usa regularmente algún medicamento y en qué cantidad); y características del cuidado del cuidador (tiempo de cuidado de la persona mayor con demencia: años, días de la semana y horas por día; experiencia anterior como cuidador; si recibe ayuda para cuidar a la persona mayor; tipo y frecuencia de la ayuda; nivel de sobrecarga y AE percibida). Para evaluar la sobrecarga, se utilizó la Escala de Zarit, traducida y validada para Brasil^(32)^. El instrumento presenta 22 ítems, con una escala de 0 a 4 puntos. El puntaje total de la escala se obtiene al sumar todos los ítems y puede variar de 0 a 88 puntos^(32)^. En este estudio, se utilizó la siguiente clasificación: sin sobrecarga (< 46 puntos), sobrecarga leve (de 46 a 56 puntos) y sobrecarga intensa (> 56 puntos)^(33)^. La AE percibida se verificó por medio de la *The General Self-efficacy Scale,* adaptada y validada para Brasil^(34)^, denominada Escala de Autoeficacia General Percibida (EAEGP). El instrumento consta de diez ítems, evaluados mediante una escala Likert de 5 puntos. En un rango de 10 a 50 puntos, cuanto mayor sea el puntaje, mayor será la percepción de AE^(34)^.

### Recopilación de datos

La recopilación de datos se realizó en el período de agosto a diciembre del 2022, y se recopilaron datos sociodemográficos, de condiciones de salud y características del cuidado del cuidador y de la capacidad de AC, utilizando la técnica de entrevista telefónica^(35)^. Antes se realizó una prueba piloto con tres CI de personas mayores de la comunidad con demencia, con características similares a las investigadas. La técnica y los instrumentos se consideraron adecuados para la recopilación de datos.

La intervención educativa se llevó a cabo en el segundo semestre del 2022 en siete tiempos (T_0_ a T_6_) (Figura 1):

**Figura 1-:** Tiempos y actividades de la intervención educativa. Cuiabá, MT, Brasil, 2023

T_0_ – Reclutamiento: mediante contacto telefónico, la investigadora informó al CI sobre la investigación, sus procedimientos y propósito y los invitó a participar en el estudio. En caso de aceptación, se leyó y se registró el Consentimiento Libre, Previo e Informado (CLPI) al cuidador, quien verbalizó su conformidad. Cada cuidador recibió, por medio de la aplicación de mensajería, una versión del CLPI con la firma de la investigadora. Posteriormente, se programó con él la mejor fecha y hora para la entrevista.

T_1_ – Preintervención: en los días y horarios programados, mediante una llamada telefónica, la investigadora recopiló de cada cuidador los datos sociodemográficos, de condiciones de salud, del cuidado brindado a la persona mayor y aplicó las escalas de sobrecarga (Escala de Zarit), de capacidad de autocuidado (EACAC) y de autoeficacia (EAEGP). Al final, se informó a los cuidadores acerca de los procedimientos de las próximas actividades de la intervención educativa.

T_2_ – Intervención: para llevar a cabo la intervención, se utilizaron cuatro vídeos como tecnología educativa. Estos vídeos fueron producidos por la propia investigadora, en la plataforma *Vyond*, con elaboración de *storyboard*, que contenía las ilustraciones/imágenes que se utilizaron para producir los vídeos, siguiendo las recomendaciones de Fleming, Reynolds y Wallace^(36)^.

Asimismo, los vídeos fueron validados en cuanto al contenido y a la apariencia por jueces expertos del ámbito de la salud y del área de la tecnología y de la comunicación, respectivamente. Para el contenido de los vídeos, se utilizaron como base teórica las cuatro fuentes de creencias de la AE de la Teoría Social Cognitiva de Albert Bandura^(37)^ (experiencia de dominio, experiencia vicaria, persuasión verbal y estados somáticos y emocionales) y una amplia revisión de la literatura sobre el AC de CI de personas mayores con demencia.

El primer vídeo duró tres minutos y cuarenta y ocho segundos y se basó en la primera fuente de la creencia de la AE: la experiencia de dominio. El escenario fue una reunión de un grupo de apoyo para cuidadores de personas mayores con demencia. El tema fue sobre el concepto de autocuidado, algunos tipos de AC y posibles formas de practicarlos. El objetivo era alentar a los CI a reflexionar sobre sus experiencias de AC como cuidadores de personas mayores con demencia. Al final, la enfermera fomentó las reflexiones sobre el tema y anunció el contenido del próximo encuentro.

El vídeo fue enviado mediante la aplicación de mensajería *WhatsApp* a cada CI. Se les informó que tenían cinco días para ver el vídeo y que podrían consultar a la investigadora, en cualquier momento, sobre problemas con los vídeos, así como aclarar dudas y hacer comentarios sobre el contenido visto. Después de cinco días, la investigadora se puso en contacto con cada cuidador, por medio de la aplicación de mensajería, para preguntar sobre el acceso al material y saber si habían visto y comprendido el contenido. En caso de respuesta afirmativa, la investigadora les preguntó sobre el contenido del vídeo para confirmar la información. A continuación, se subió el segundo vídeo.

T_3_ – Intervención: basado en la segunda fuente de creencia de la AE —la experiencia vicaria—, el objetivo de este vídeo fue animar al CI, por medio de la observación de la experiencia de otros cuidadores de personas mayores con demencia, a reflexionar sobre la capacidad de cuidarse a sí mismo y ayudarlo a darse cuenta de que puede beneficiarse de modelos que contribuyan a mejorar su autocuidado. Para ello, utilizando el mismo escenario que el vídeo anterior, la enfermera presentó a los CI el testimonio de tres cuidadores sobre sus experiencias de autocuidado y los beneficios de esta práctica para la calidad de vida y la salud. La duración de este vídeo fue de siete minutos y cincuenta y siete segundos, y el tema fue las experiencias de AC de cuidadores y los beneficios de esta práctica para la calidad de vida y la salud. Como lo había hecho anteriormente, cinco días después, la investigadora repitió los procedimientos mencionados y envió el tercer vídeo.

T_4_ – Intervención: el tercer vídeo se basó en la tercera fuente de creencia de AE —la persuasión verbal—, y el objetivo fue incentivar y estimular al CI a afrontar las dificultades para llevar a cabo el autocuidado. El tema fue evidencias científicas sobre los beneficios que las conductas de AC brindaron a CI de personas mayores que viven con demencia. En este vídeo, con duración de cuatro minutos y once segundos, basado en evidencias científicas, la enfermera abordó los beneficios que obtuvieron los CI de personas mayores que viven con demencia mediante las conductas de autocuidado. Cinco días después, la investigadora repitió los procedimientos anteriores y envió el cuarto vídeo.

T_5_ – Intervención: basado en la cuarta fuente de creencias de AE —estados somáticos y emocionales—, este vídeo tuvo como objetivo capacitar a los CI para aprender algunas estrategias de afrontamiento a las dificultades experimentadas para llevar a cabo el autocuidado, y el tema fue estrategias para que el CI practique el AC. El vídeo tuvo una duración de cuatro minutos y cincuenta y un segundos, y en él la enfermera ejemplificó algunas estrategias de autocuidado, considerando las dificultades relatadas por los cuidadores. El vídeo termina con la enfermera despidiéndose de los CI y poniéndose a disposición para aclarar sus dudas. Cinco días después de los procedimientos anteriores, la investigadora informó que esta etapa había terminado y programó con cada uno de los CI el día y hora para realizar la posintervención.

T_6_ – Posintervención: la investigadora realizó, mediante llamada telefónica, una nueva medida de la capacidad de AC, aplicando la EACAC.

### Análisis de datos

El análisis de datos se realizó utilizando el programa STATA, versión 16.1. El análisis descriptivo se presentó en tablas y gráficos, con frecuencia absoluta y relativa. Para comparar los datos antes y después de la intervención, se realizó la prueba de normalidad de los datos y la presentación gráfica con *boxplot*. Posteriormente, se realizó una prueba no paramétrica de Wilcoxon *Rank-Sum* y una prueba t pareada y cálculo de media, desviación estándar, mediana, mínima y máxima. Se consideró un nivel de significancia de 0,05.

## Aspectos éticos

El proyecto de investigación fue aprobado bajo el dictamen 4.622.301 por el Comité de Ética en Investigación del Hospital Universitário Júlio Müller y fue registrado en el Registro Brasileño de Ensayos Clínicos (ReBEC) bajo el número RBR-7gfvn3y.

## Resultados

De los 17 CI de personas mayores con demencia encuestados, la mayoría son mujeres (88,2%) con más de 11 años de educación (58,8%), el 41,2% tiene entre 50 y 59 años, y el 47% está casada o en unión estable. Respecto a la ocupación, el 35,3% no trabaja. En cuanto a los ingresos, un 29,4% recibe hasta un salario mínimo y otro 29,4% no tiene ninguno. Casi la mitad de los ingresos de los CI (41,7%) proviene de la jubilación, y del 41,7% proviene del trabajo. La mayoría de los cuidadores son hijo/hija de la persona mayor (76,5%), el 88,2% vive con ella, el 53,3% durante 15 años o más.

Respecto al estado de salud, el 41,2% de los CI autoevalúan su salud como regular. La mayoría (88,2%) no fuma, y el 64,7% no consume bebidas alcohólicas. Respecto a los problemas de salud, solo el 23,6% afirma tener tres o más, y los principales problemas reportados son los cardiovasculares (40,0%), psiquiátricos (40,0%) y endocrinos (30,0%). Una gran parte (52,9%) declara no tomar medicamentos, y el 29,4% toma de dos a cuatro medicamentos regularmente.

Respecto a las actividades como cuidador, un 29,4% lleva entre 5 y 9 años cuidando a la persona mayor, y otro 29,4% lleva 10 años o más cuidándola. La mayoría niega experiencia previa como cuidador (52,9%). La mayoría refiere brindar atención todos los días de la semana (76,4%), la mayoría durante las 24 horas del día (88,2%). El mayor número de CI (76,5%) afirma recibir algún tipo de ayuda con la persona mayor, y el 53,8% refiere que el tipo de ayuda que más recibe es en el cuidado de la persona mayor. Respecto al nivel de sobrecarga, evaluado mediante la Escala de Zarit, la mayoría de los cuidadores (70,6%) fue clasificada en la categoría sin sobrecarga. La mayoría (94,1%) obtuvo un buen puntaje en la medida de AE percibida evaluada por la EAEGP.

La evaluación de la capacidad de AC antes y después de la intervención se presenta en la Figura 2.

**Figura 2-:** Comparación de la capacidad de autocuidado antes y después de la intervención educativa. Cuiabá, MT, Brasil, 2023

En la Tabla 1 y la Figura 3 se presenta el análisis comparativo del efecto de la intervención educativa sobre la capacidad de AC de los CI. En la Tabla 1 constan los valores antes y después de la intervención en lo que se refiere a la media, la mediana, la desviación estándar, la mínima y la máxima y el valor de *p*.

**Tabla 1-:** Análisis comparativo del efecto de la preintervención y posintervención educativa. Cuiabá, MT, Brasil, 2023

**Variable**	**Media**	**Mediana**	DP*	**Mín./Máx.** ^†^	p **valor**
**Capacidad de autocuidado**					**0,029** ^‡^
Preintervención	95,00	091,00	14,40	68;118	
Posintervención	102,11	105,00	13,39	63;116	

*DE = Desviación estándar; ^†^Mín./Máx. = Mínima y máxima; ^‡^Prueba pareada de Wilcoxon

En el *boxplot* (Figura 3) se puede comprobar la diferencia de las medianas, la dispersión de los datos y las diferencias entre el 3.^er^ y el 1.^er^ cuartiles en la comparación antes y después de la intervención.

**Figura 3-:** *Boxplot* del análisis comparativo de la capacidad de autocuidado antes y después de la intervención. Cuiabá, MT, Brasil, 2023

*EACAC = Escala para Evaluar la Capacidad de Autocuidado

Con base en el análisis comparativo intraindividual de la capacidad de AC, la Figura 4 muestra los puntajes en la comparación antes y después de la intervención.

**Figura 4-:** Comparación intraindividual de los puntajes de la Escala de Evaluación de la Capacidad de Autocuidado después de la intervención educativa. Cuiabá, MT, Brasil, 2023

*EACAC = Escala para Evaluar la Capacidad de Autocuidado

## Discusión

El principal hallazgo de este estudio muestra que la intervención fue efectiva para aumentar la capacidad de AC de los CI, principalmente con relación al cambio en los puntajes de aquellos que se encontraban en la categoría buena.

Un punto que tener en cuenta en el análisis es que la mayoría de los participantes de la muestra de este estudio tienen condiciones que pueden contribuir al AC. Aunque cuidan a personas mayores durante un largo período de tiempo, brindándoles atención todos los días de la semana y durante las 24 horas, fueron clasificados como sin sobrecarga. Quizá porque la mayoría recibe algún tipo de ayuda con la persona mayor, tanto en los cuidados como en el aspecto económico. La literatura evidencia que en estas condiciones el AC puede ser mejor practicado, como en el estudio sobre los factores relacionados con la sobrecarga y el AC de la hipertensión en 68 cuidadores familiares de personas mayores, lo que puso de manifiesto que los cuidadores que recibían ayuda de otra persona para cuidar a la persona mayor presentaron un mayor puntaje en el dominio grado de confianza respecto a su condición de hipertenso, cuando se comparan con los que no recibían ayuda. Cuanto más prolongado sea el tiempo de cuidado brindado, menor será el puntaje del dominio medidas de gestión del AC durante la descompensación de la presión^(38)^.

Asimismo, se debe considerar que los participantes de este estudio, tras la intervención, comenzaron a presentar mejores puntajes en la capacidad de AC, lo que aumentó los valores porcentuales de las categorías muy buena y excelente. Este resultado probablemente se produjo porque ya tenían un buen puntaje de la AE antes de la intervención.

La literatura muestra que la AE es un determinante del comportamiento de AC y que puede influir en la decisión de cuidarse a uno mismo^(29)^. Los puntajes más altos de AE se asocian con mejoras en la salud general, en la calidad de vida, en la salud mental y la autoestima, en el funcionamiento social y en la capacidad de AC con asertividad en la toma de decisiones^(39)^. Además, una mayor AE puede estar relacionada con un mejor desempeño en las conductas de AC^(40)^, es decir, cuanto mayor sea la AE de un individuo, mayor será el esfuerzo y empeño para lograr sus objetivos y para practicar el autocuidado^(41)^. Las creencias de AE aumentan la intensidad del esfuerzo que las personas ponen al llevar a cabo una determinada acción, lo que puede influir en sus decisiones y modificar la forma en que afrontan las dificultades que experimentan^(28)^.

Además, la intervención basada en la AE puede haber contribuido a mejorar aún más la capacidad de AC de los CI. El hecho de que la intervención trabajara sobre las fuentes de AE con el objetivo de influir en el aumento del AC pudo haber mejorado la AE de estos cuidadores, lo que, a su vez, contribuyó al aumento del AC. Parece que alentar a las personas a pensar sobre sus experiencias de AC, a reflexionar sobre los beneficios del AC para sus vidas, compartir las experiencias positivas de AC de otros cuidadores y alentarlos a adoptar medidas de AC influyó en las conductas de AC por parte de los cuidadores en este estudio. Esto también se encontró en estudios que utilizaron el constructo de la AE en intervenciones educativas para mejorar las conductas de AC de pacientes sometidos a prostatectomía radical^(42)^, pacientes en hemodiálisis^(43)^ y mujeres embarazadas^(44)^.

Asimismo, se puede considerar que el nivel de educación de los cuidadores en esta investigación favoreció el incremento de la capacidad de AC de estos cuidadores, ya que la mayoría contaba con 11 o más años de estudio. La literatura ha demostrado la asociación entre el nivel de educación y las conductas de AC^(45-47)^, demostrando que puede ser un condicionante en la forma como los individuos se involucran y realizan acciones para el AC, pues se refleja en su capacidad para comprender los contenidos y las pautas de las intervenciones educativas^(46,48)^.

El análisis comparativo de la capacidad de AC presentada en el *boxplot* reafirma que hubo un aumento en la capacidad de AC de los cuidadores y en la efectividad de la intervención educativa, una vez que se observa un cambio significativo en la distribución de puntajes y en la dispersión de los datos antes y después de la intervención.

Sin embargo, aunque los resultados son positivos, se debe tener en cuenta que, en el análisis comparativo intraindividual de la capacidad de AC, hubo mantenimiento y disminución en el puntaje de las respuestas de algunos participantes de la investigación después de la intervención educativa. Esto también pudo haber sucedido porque la intervención se basó en las fuentes de AE. Cada fuente tiene como objetivo motivar al individuo a reflexionar sobre su capacidad AE para adoptar determinadas conductas. Según Bandura^(37)^, el individuo tiene la capacidad de reflexionar sobre el valor y el significado de sus acciones y, si es necesario, realizar ajustes. Cada individuo, a partir de una misma experiencia, procesa cognitivamente las fuentes de AE, y cada interpretación puede llevar a diferentes creencias de eficacia^(49)^.

Las fuentes de creencias de AE pueden influenciar al individuo en la percepción de sus acciones y actitudes. Si estas fuentes de AE indican que sus acciones/actitudes están en línea con las expectativas, la persona puede percibir que está actuando de manera apropiada. Pero si indican dificultades, fallos o desajustes, la persona puede percibir la necesidad de ajustarlo^(27)^.

En ese sentido, es posible que estos cuidadores, al ser expuestos a las situaciones trabajadas en cada vídeo, tuvieran la oportunidad de reflexionar sobre su AC y el de otras personas. Por ejemplo, cuando las fuentes de AE “Experiencia de dominio” y “Experiencia vicaria” se abordaron en los vídeos para alentar a los cuidadores a reflexionar sobre sus experiencias de AC, puede que aquellos cuyos puntajes disminuyeron después de la intervención hayan reconsiderado sus conductas de AC. Quizá la intervención haya influido en la AE percibida (percepción de la propia capacidad) de los CI y los haya motivado a ajustar su actitud, llegando a la conclusión de que lo que practicaban no era AC o que su AC era insuficiente.

Este estudio presenta algunas limitaciones. El tamaño de la muestra reduce la posibilidad de generalizar los resultados obtenidos y su aplicabilidad a otras situaciones. Además, con una muestra pequeña, la variabilidad en los resultados puede ser alta. Sin embargo, las pruebas estadísticas demostraron que, en esta población, no ocurrió esta variabilidad; por el contrario, hubo una disminución en la dispersión de los datos. Esto mejora la validez interna del estudio, sugiriendo que las diferencias observadas antes y después pueden deberse a la intervención y no a otros factores. De lo contrario, una menor variabilidad significa que los resultados son más confiables.

Los resultados de este estudio suponen un avance en el conocimiento científico sobre el cuidado que los CI brindan a las personas mayores, específicamente a aquellas que viven con demencia. El AC es un elemento fundamental en este cuidado, y las intervenciones educativas que contribuyan a sus conductas son muy importantes, especialmente para los profesionales de enfermería que tienen la oportunidad de trabajar de cerca con esta población. Existe, por lo tanto, potencial para que la enfermería practique la educación de CI para contribuir al desarrollo del AC, mitigando la sobrecarga relacionada con el cuidado de personas mayores con demencia con beneficios para su calidad de salud y vida.

Se recomienda que se realicen más estudios de intervención sobre el AC de CI de personas mayores con demencia, aplicados a muestras más grandes, que permitan la generalización de los resultados. Asimismo, se sugiere desarrollar estudios similares, pero de naturaleza longitudinal, con el fin de supervisar y proporcionar resultados a lo largo del tiempo, además de investigaciones con diseño de ensayo clínico aleatorizado, con muestras más amplias.

## Conclusión

La intervención educativa basada en la AE fue efectiva para incrementar la capacidad de AC de CI de personas mayores que viven con demencia, una vez que obtuvo valores estadísticamente significativos en la posintervención en comparación con la preintervención. Este resultado probablemente se produjo porque la intervención educativa se llevó a cabo mediante vídeos basados en las fuentes de creencias de AE con el objetivo de promover el aumento del AC. Además, pueden haber contribuido el buen nivel educativo de los participantes, el hecho de que recibieran algún tipo de ayuda para cuidar a la persona mayor, que no tuvieran sobrecarga y que ya tuvieran un buen puntaje de AE antes de la intervención.

## Data Availability

Todos los datos generados o analizados durante este estudio están incluidos en este artículo publicado.
